# Quantitative Proteomics Reveals Dynamic Interactions of the Minichromosome Maintenance Complex (MCM) in the Cellular Response to Etoposide Induced DNA Damage*[Fn FN2]

**DOI:** 10.1074/mcp.M115.048991

**Published:** 2015-05-11

**Authors:** Romain Drissi, Marie-Line Dubois, Mélanie Douziech, François-Michel Boisvert

**Affiliations:** From the ‡Department of Anatomy and Cell Biology, Université de Sherbrooke, 3201 Jean-Mignault, Sherbrooke, Québec, J1E 4K8, Canada

## Abstract

The minichromosome maintenance complex (MCM) proteins are required for processive DNA replication and are a target of S-phase checkpoints. The eukaryotic MCM complex consists of six proteins (MCM2–7) that form a heterohexameric ring with DNA helicase activity, which is loaded on chromatin to form the pre-replication complex. Upon entry in S phase, the helicase is activated and opens the DNA duplex to recruit DNA polymerases at the replication fork. The MCM complex thus plays a crucial role during DNA replication, but recent work suggests that MCM proteins could also be involved in DNA repair. Here, we employed a combination of stable isotope labeling with amino acids in cell culture (SILAC)-based quantitative proteomics with immunoprecipitation of green fluorescent protein-tagged fusion proteins to identify proteins interacting with the MCM complex, and quantify changes in interactions in response to DNA damage. Interestingly, the MCM complex showed very dynamic changes in interaction with proteins such as Importin7, the histone chaperone ASF1, and the Chromodomain helicase DNA binding protein 3 (CHD3) following DNA damage. These changes in interactions were accompanied by an increase in phosphorylation and ubiquitination on specific sites on the MCM proteins and an increase in the co-localization of the MCM complex with γ-H2AX, confirming the recruitment of these proteins to sites of DNA damage. In summary, our data indicate that the MCM proteins is involved in chromatin remodeling in response to DNA damage.

DNA replication during the S phase necessitates that the entire genome be duplicated with the minimum of errors. Thousands of replication forks are involved in this process and they must be coordinated to ensure that every section of DNA is only replicated once. Errors in DNA replication are likely to be a major cause of the genetic instability that can lead to cancer (1). Cells are able to prevent duplicate replication of DNA by having a distinct stage that occurs during the G1 phase when replication origins are “licensed” for replication, a process that involves the preloading of several proteins involved in DNA replication (2). As DNA is replicated at each origin, these proteins are removed, thereby ensuring that each origin fires only once during each S phase. DNA damage response kinases activated by the stalled forks prevent the replication machinery from being activated in new chromosome domains, indicating a tight relationship between the DNA damage response and the DNA replication pathways (3, 4).

The first step of the replication licensing mechanism is the loading of the minichromosome maintenance (MCM)^1^ proteins on to replication origins along with origin recognition complex proteins, Cdt6 and Cdt1 (5). The eukaryotic MCM complex consists of six paralogs that form a heterohexameric ring. All eukaryotic organisms possess six homologous proteins (MCM2-MCM7) that form a heterohexameric ring that belong to the family of AAA+ (ATPase associated with various cellular activities) proteins and share similarities to other hexameric helicases (6). Even though additional MCM proteins have been identified in higher eukaryotes, the MCM2-MCM7 complex remains the prime candidate for the role of replicative helicase (7). MCM2–7 is required for both initiation and elongation of DNA replication, with its regulation at each stage being an essential player of eukaryotic DNA replication (8). As a critical mechanism to ensure only a single round of DNA replication, the loading of additional MCM2–7 complexes onto origins of replication is inactivated by redundant mechanisms after passage into S phase (9).

The MCM complex plays a crucial role in determining the replication potential of cells, but recent work suggests that MCM proteins are not only targets of the S-phase checkpoints, but they also interact directly with components of the checkpoint and repair pathways (10, 11). In *yeast*, temperature sensitive MCM cells at restrictive temperature contain numerous foci recognized by the phosphorylated histone H2AX antibody (12), suggesting a role in the repair of DNA double-strand breaks. Although, in principle, only two DNA helicase activities are required to establish a bidirectional replication fork from each origin, a relatively large excess of MCM complexes are loaded at origins of replication and distributed along the chromatin (13). Their function is not well understood, and most of them are displaced from the DNA during S-phase, apparently without having played an active role in DNA replication. The “MCM paradox” refers to the fact that, at least in yeast, *Xenopus*, Drosophila, and mammalian cells, it is possible to reduce the concentration of MCM proteins by more than 90% without impairing DNA replication (14–18) and also refers to the observation that the majority of MCM complexes do not localize to the sites of DNA synthesis in mammalian cells, further suggesting a potential role for the MCM proteins beyond DNA replication.

Using a combination of stable isotope labeling with amino acids in cell culture (SILAC)–based quantitative proteomics (19) with immunoprecipitation of green fluorescent protein (GFP)-tagged fusion proteins (20), we identified differences in protein binding partners with the MCM complex following DNA damage. Stable cell lines expressing GFP-tagged MCM2 and MCM5 were used in immunoprecipitation experiments from cells that were either mock treated, or treated with Etoposide for 15, 60, and 240 min. Etoposide is an antitumor drug that stabilizes a covalent complex between the DNA topoisomerase II and DNA by interfering with the cleavage-ligation reaction of the topoisomerase (21). This revealed specific interaction between the MCM complex and several proteins such as Nucleophosmin, BAG2, UPP1, and HDAC10. Interestingly, the MCM complex showed dynamic changes in interaction with Importin7 and the histone chaperone ASF1, and a decrease in interaction with the Chromodomain helicase DNA binding protein 3 (CHD3) resulting from the treatment with etoposide. This increase in interaction with ASF1 was followed by an enrichment of histone proteins, suggesting a novel role for the MCM proteins in histone deposition on chromatin following DNA damage.

## EXPERIMENTAL PROCEDURES

### 

#### 

##### Cell Culture and Stable Cell Lines

U2OS and U2OS Flp-In T-Rex cells were grown as adherent cells in Dulbecco's modified eagle medium (DMEM) depleted of arginine and lysine (Life Technologies, Carlsbad, CA A14431–01) supplemented with 10% dialyzed fetal bovine serum (Invitrogen, Carlsbad, CA 26400–044), 100 U/ml penicillin/streptomycin and 2 mm GlutaMax. Arginine and lysine were added in either light (Arg0, Sigma, A5006; Sigma-Aldrich (St-Louis, MO) Lys0, Sigma, L5501), medium (Arg6, Cambridge Isotope Laboratories, Inc. (Tewksbury, MA) (CIL), CNM-2265; Lys4, CIL, DLM-2640), or heavy (Arg10, CIL, CNLM-539; Lys8, CIL, CNLM-291) form to a final concentration of 28 μg/ml for arginine and 49 μg/ml for lysine. l-proline was added to a final concentration of 10 μg/ml to prevent arginine to proline conversion (see supplemental Fig. 1). Proteins were tested for >99% incorporation of the label after six passages by mass spectrometry (data not shown). U2OS stable cell lines were generated by transfecting pgLAP1 plasmids containing the cDNA of interest along with pOG44, the plasmid expressing the Flp-recombinase. Cells were then cultured with the addition of 150 μg/ml Hygromycin B and 15 μg/ml Blasticidine-HCl. For induction of DNA damage, the topoisomerase II inhibitor etoposide (#E1383, Sigma-Aldrich) was used at the indicated concentrations for 1 h, followed by wash with PBS and fresh normal media added.

##### Cloning and Generation of Plasmids

MCM2, MCM5, and ASF1A were amplified by PCR using oligonucleotides that included BP recombination sites from a cDNA library generated by RT-PCR with an oligo-dT following isolation of mRNA by Trizol on U2OS cells. The PCR product was then incorporated by recombination into pDONR 221 (Life Technologies) using BP recombinase and subsequently cloned into pgLAP1 using Gateway cloning (Life Technologies) (22).

##### Antibodies

The following antibodies were used: anti-GFP (Roche 11814460001), anti-MCM2 (Rabbit polyclonal, Abcam #Ab31159, Cambridge, MA), anti-MCM5 (Rabbit monoclonal, Abcam #Ab75975), anti-ASF1 (Rabbit monoclonal, Cell Signaling #C6E10, Danvers, MA), anti-γH2AX (Rabbit polyclonal, Santa Cruz #sc-101696, Dallas) and anti-H2B (Rabbit polyclonal, Cell Signaling #2722). Secondary antibodies used were anti-mouse IgG-HRP (Goat polyclonal, Santa Cruz #sc-2005, Dallas, TX) and anti-rabbit IgG-HRP (Goat polyclonal, Santa Cruz #sc-2004).

##### Immunofluorescence Microscopy

Cells were cultured on glass coverslips in six-well plates and fixed with 1% paraformaldehyde in PBS for 10 min at room temperature. Fixed cells were washed with PBS and permeabilized using 0.5% Triton X-100 in PBS for 10 min followed by washing in PBS. Coverslips were incubated with primary antibodies diluted in PBS for 1 h, and then washed once in 0.1% Triton X-100 in PBS and twice in PBS. Primary antibodies were detected with Alexa 488 or 546 conjugated secondary antibodies (Molecular Probes, Carlsbad, CA) on coverslips for 1 h in PBS. DNA was counterstained with 4.6-diamidino-2-phenylindole (DAPI). After wash with PBS, the coverslips were mounted on glass slides on a drop of Shandon Immuno-Mount (Thermo Scientific).

##### GFP-Immunoprecipitation from SILAC Labeled U2OS Cells

Cells grown in each SILAC medium were harvested separately by scraping in PBS and the cell pellets were lysed in IP buffer (1% Triton X-100, 10 mm Tris pH 7.4, 150 mm NaCl, Roche Complete Protease Inhibitor Mixture) for 10 min on ice. The lysates were then centrifuged for 10 min at 13,000 × *g* at 4^°^C and equal amount of proteins were incubated with GFP-trap agarose beads from ChromaTek (Martinsried, Germany) for 2 h at 4°C. Beads were then washed three times with IP buffer and twice with PBS. After the last wash, the beads from the three SILAC conditions were resuspended in PBS and combined before removing the remaining PBS. The beads were then resuspended LDS sample buffer and the samples processed for in-gel digestion.

##### Gel Electrophoresis and In-gel Digestion

For each time point, proteins were reduced in 10 mm DTT and alkylated in 50 mm iodoacetamide prior to boiling in loading buffer, and then separated by one-dimensional SDS-PAGE (4–12% Bis-Tris Novex mini-gel, Life Technologies) and visualized by Coomassie staining (Simply Blue Safe Stain, Life Technologies). The entire protein gel lanes were excised and cut into 8 slices each. Every gel slice was subjected to in-gel digestion with trypsin (23). The resulting tryptic peptides were extracted by 1% formic acid, then 100% acetonitrile, lyophilized in a speedvac, and resuspended in 1% formic acid.

##### LC-MS/MS

Trypsin digested peptides were separated using a Dionex Ultimate 3000 nanoHPLC system. Ten microliters of sample (a total of 2 μg) in 1% (v/v) formic acid was loaded with a constant flow of 4 μl/min onto an Acclaim PepMap100 C18 column (0.3 mm id × 5 mm, Dionex Corporation, Sunnyvale, CA). After trap enrichment peptides were eluted off onto a PepMap C18 nano column (75 μm × 50 cm, Dionex Corporation) with a linear gradient of 5–35% solvent B (90% acetonitrile with 0.1% formic acid) over 240 min with a constant flow of 200 nl/min. The HPLC system was coupled to an OrbiTrap QExactive mass spectrometer (Thermo Fisher Scientific Inc) via an EasySpray source. The spray voltage was set to 2.0 kV and the temperature of the column was set to 40 °C. Full scan MS survey spectra (*m*/*z* 350–1600) in profile mode were acquired in the Orbitrap with a resolution of 70,000 after accumulation of 1,000,000 ions. The ten most intense peptide ions from the preview scan in the Orbitrap were fragmented by collision induced dissociation (normalized collision energy 35% and resolution of 17,500) after the accumulation of 50,000 ions. Maximal filling times were 250 ms for the full scans and 60 ms for the MS/MS scans. Precursor ion charge state screening was enabled and all unassigned charge states as well as singly, seven and eight charged species were rejected. The dynamic exclusion list was restricted to a maximum of 500 entries with a maximum retention period of 40 s and a relative mass window of 10 ppm. The lock mass option was enabled for survey scans to improve mass accuracy. Data were acquired using the Xcalibur software.

##### Quantification and Bioinformatics Analysis

Data were processed, searched, and quantified using the MaxQuant software package version 1.4.1.2 as described previously (24) employing the Human Uniprot database (16/07/2013, 88,354 entries). The settings used for the MaxQuant analysis were: 2 miscleavages were allowed; fixed modification was carbamidomethylation on cysteine; enzymes were Trypsin (K/R not before P); variable modifications included in the analysis were methionine oxidation and protein N-terminal acetylation. A mass tolerance of 7 ppm was used for precursor ions and a tolerance of 20 ppm was used for fragment ions. The re-quantify option was selected to calculate the ratio for isotopic patterns not assembled in SILAC pairs as often observed during pulldown experiments (25). To achieve reliable identifications, all proteins were accepted based on the criteria that the number of forward hits in the database was at least 100-fold higher than the number of reverse database hits, thus resulting in a false discovery rate (FDR) of less than 1%. A minimum of 2 peptides were quantified for each protein. Protein isoforms and proteins that cannot be distinguished based on the peptides identified are grouped and displayed on a single line with multiple accession numbers (see supplementary tables 1–4).

##### Data Submission

The mass spectrometry data have been deposited to the ProteomeXchange Consortium (http://proteomecentral.proteomexchange.org) via the PRIDE partner repository with the data set identifier PXD001949.

## RESULTS

**Fig. 1.**
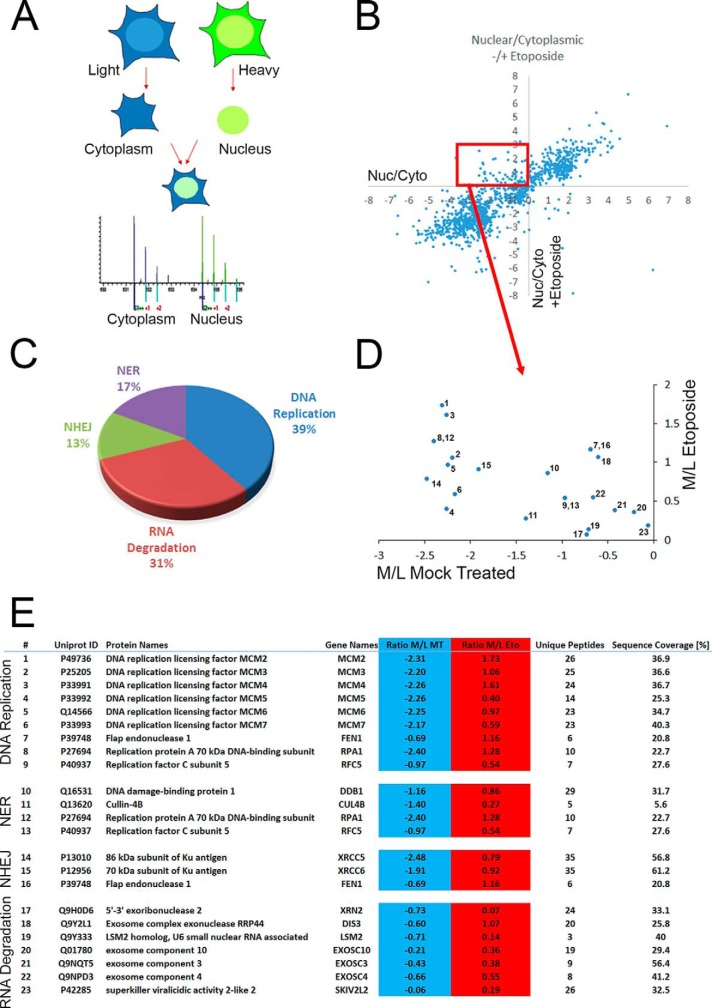
**Protein subcellular relocalisation following DNA damage.** *A*, Schematic diagram of spatial proteomics to quantify cytoplasmic and nuclear localization. Proteins from SILAC-labeled cells are biochemically fractionated into cytoplasmic and nuclear fraction which are then recombined prior to identification and quantification by mass spectrometry. *B*, Nuclear/Cytoplasmic ratios of proteins from mock treated cells (*x* axis) compared with proteins treated with 50 μm Etoposide for 1 h (*y* axis). *C*, KEGG pathway enrichment analysis on the 93 proteins showing a cytoplasmic to nuclear relocalisation following DNA damage. *D*, Numeration of the 23 proteins identified in the KEGG pathway enrichment according to the table (*E*) showing the list of proteins classified in each KEGG pathways.

Using a quantitative proteomic approach to measure the protein content in different cellular fractions, we utilized a technique called spatial proteomics that measures the cellular distribution of thousands of proteins using a combination of cellular fractionation and mass spectrometry (26). This method involves first culturing cells with SILAC medium to ensure proteins are fully labeled (19). The SILAC incorporated cells are then separated into fractions, for example, the cytoplasm and nucleus which are recombined such that each fraction has a distinct isotope signature (Fig. 1*A*). The labeling thus allows quantification of the relative abundance of peptides originating from subcellular fraction and has been used to study the relative distribution of the proteome between the cytoplasm, nucleus, and nucleolus (26).

In order to identify proteins potentially involved in the cellular response to DNA damage, we used the spatial proteomics method to identify proteins whose localization would show a change in their subcellular distribution following treatment with etoposide, a topoisomerase II inhibitor causing double-strand breaks (Fig. 1*B*, supplemental Table S1*a*). Out of 1933 proteins identified, 93 proteins showed a relocalization from the cytoplasmic fraction to the nuclear fraction (supplemental Table S1*b*). To identify whether proteins with common known cellular functions were specifically affected in response to DNA damage, enrichment in Kegg pathway annotations of the 93 proteins showing a change in cytoplasmic to nuclear localization was analyzed (Fig. 1*C*, supplemental Table S1*c*). We found four different pathways significantly enriched (*p* values <0.01) that includes 23 proteins within the group of 93 proteins (Fig. 1*D* and 1*E*). The pathways involved were DNA replication, RNA degradation, nucleotide excision repair, and nonhomologous end-joining (supplemental Table S1*c*). Of the proteins identified involved in DNA replication, the six proteins forming the MCM complex were identified (Fig. 1*E*).

**Fig. 2.**
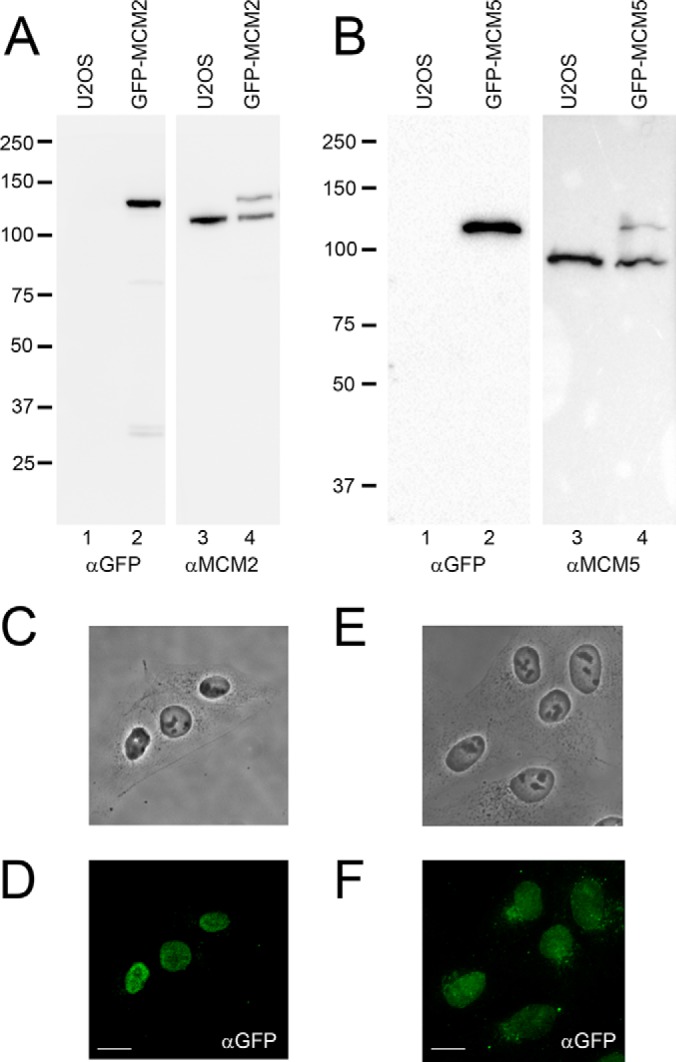
**Generation of inducible stable cell lines expressing GFP-MCM2 and GFP-MCM5.** *A*, and *B*, GPF-MCM2 and GFP-MCM5 were generated by Flp-In recombination into U2OS cells at a specific integration site. Whole cell extracts from either non-induced U2OS cells (*A* and *B*, lane 1 and 3), or from cells induced for expression of GFP-MCM2 (A, lane 2 and 4) or GFP-MCM5 (*B*, lane 2 and 4) were separated by SDS-PAGE and immunoblotted with a GFP antibody (*A* and *B*, lane 1–2), with a MCM2 antibody (*A*, lane 3–4) and with a MCM5 antibody (B, lane 3–4) to confirm expression of the GFP-tagged proteins. *C–F*) Cells induced for expression of GFP-MCM2 (*C–D*) and GFP-MCM5 (*E–F*) were fixed and labeled for immunofluorescence using a GFP antibody (*D* and *F*) and cells were visualized by phase contrast (*C–E*). Scale bar = 30 μm.

To further characterize the involvement of the MCM complex in the cellular response to DNA damage, we decided to identify proteins interacting with different MCM proteins in the absence and presence of DNA damage. First, we generated inducible stable cell lines expressing MCM2 and MCM5 at close to endogenous levels. These cell lines were generated using LAP1-tagged wild type MCM2 and MCM5 with a GFP at the N-terminal (Fig. 2) by flp-in recombination into U2OS cells that have a single integration site. The exogenous GFP-MCM2 and GFP-MCM5 proteins were found in similar levels compared with the endogenous proteins (Fig. 2*A* and 2*B*, lanes 4). In fact, expression of the exogenous protein appear to reduce the amount of the endogenous protein expressed, suggesting a possible mechanism regulating the total amount of MCM proteins present in the cell (Fig. 2*A* and 2*B*, compare lanes 3 and 4 and supplemental Fig. 2). The GFP-tagged proteins is mostly nuclear as demonstrated by immunofluorescence microscopy (Fig. 2*C* and 2*D*).

**Fig. 3.**
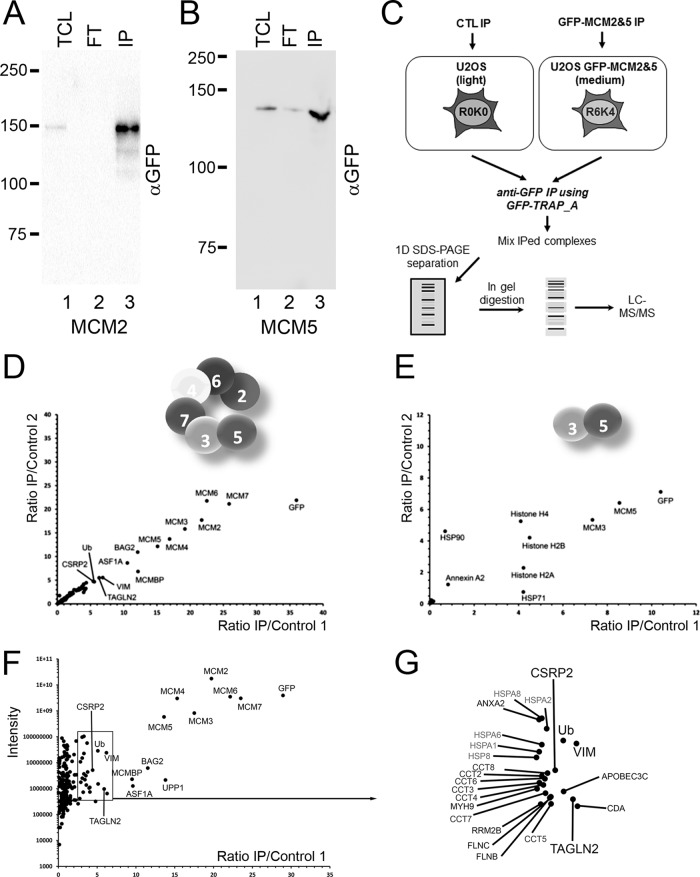
**AP-MS of GFP-MCM2 and GFP-MCM5 to identify interacting proteins.** *A*, GFP tagged MCM2 and *B*, MCM5 proteins were immunoprecipitated using GFP-TRAP agarose beads, ensuring a near depletion of all the tagged MCM protein from the cell lysate. *C*, SILAC-labeled cells were used for comparison of GFP-based immunoprecipitates from uninduced cells (light) with doxycycline-induced cells (medium). Immunoprecipitates were combined, separated by SDS-PAGE, each gel lane cut into eight slices prior to in-gel digestion with trypsin. The extracted peptides were analyzed by LC-MS/MS. The M/L ratio of two independent experiments for GFP-MCM2 (*D*) and GFP-MCM5 (*E*) were plotted and proteins with ratio above the level of the contaminants were identified. F, The average M/L ratios of the two GFP-MCM2 AP-MS experiments were plotted *versus* the total intensities. *G*, A zoom over the boxed region in *F*, for identification of proteins with ratios above the contaminants.

Next, we investigated which proteins were interacting with MCM2 and MCM5 by performing SILAC-based quantitation of interaction partners for each of those proteins. GFP tagged MCM2 and MCM5 proteins were immunoprecipitated using GFP-TRAP agarose beads, ensuring a near depletion of all the tagged MCM protein from the cell lysate (Fig. 3*A*). We compared GFP-based immunoprecipitates from uninduced cells (light) with doxycycline-induced cells (medium) (Fig. 3*C*). This experimental design allowed discrimination between specific interactions and contaminant proteins. Analysis of the MCM2 experiment identified all the proteins from the MCM complex (MCM2–7, Fig. 3*D*), along with several other proteins (Fig. 3*F*–3*G* and supplemental Fig. 3), indicating that the GFP-MCM2 proteins is properly assembled within the MCM complex. Among the proteins identified interacting specifically with the MCM complex, we identified with a relatively high ratio of enrichment other known interactors such as MCM binding protein (MCMBP), as well as several chaperones involved in protein folding such as heat shock proteins and protein from the T-complex 1 or CCT complex (Fig. 3*F*, supplemental Table S2*a*). Such proteins are often identified interacting with exogenously expressed proteins (27), and are thus most likely to be specific interactions to help folding the excess protein, but are less interesting in understanding the normal interactions of the endogenous proteins. On the other hand, analysis of the MCM5 experiment only identified MCM3 and histones (Fig. 3*E*, supplemental Fig. S2*b* and supplemental Fig. 3), indicating that GFP-MCM5 is only interacting with his direct binding partner (28) and is apparently not forming a functional MCM complex.

**Fig. 4.**
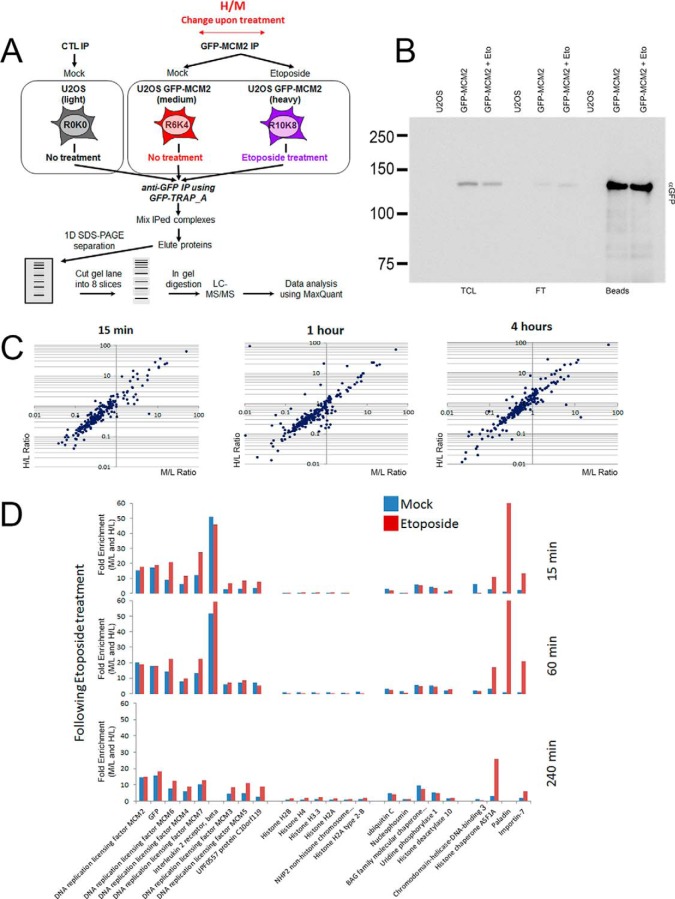
**Dynamic interactions of the MCM complex following DNA damage.** *A*, A schematic representation of the SILAC based AP-MS experiment to identify and quantify proteins interacting with GFP-MCM2 following treatment with etoposide at 50 μm for 1 h. SILAC-labeled cells were used for comparison of GFP-based immunoprecipitates from uninduced cells (light), of DMSO (mock) treated cells induced for GFP-MCM2 expression (medium) and of etoposide treated cells induced for GFP-MCM2 expression (heavy). Immunoprecipitates for each experiments were combined, separated by SDS-PAGE, each gel lane cut into 8 slices prior to in-gel digestion with trypsin. The extracted peptides were analyzed by LC-MS/MS. *B*, Total cell lysates (TCL) and immunoprecipitates from control, GFP-MCM2 mock treated and GFP-MCM2 cells treated with 50 μm etoposide were separated by SDS-PAGE and immunoblotted with a GFP antibody. *C*, Ratios of proteins interacting with GFP-MCM2 from mock treated cells (M/L, *x* axis) *versus* cells treated with etoposide for 1 h at 50 μm and allowed to recover for 15, 60, and 240 min (H/L, *y* axis). *D*, Identification of proteins identified with the M/L ratios (blue) and the H/L ratios (red) above contaminants at 15, 60, and 240 min following treatment with etoposide.

To characterize protein interactions that are regulated by DNA damage, we decided to continue with only GFP-MCM2 because it was the only protein to be incorporated in the complete MCM complex (Fig. 3). In order to identify interactions that are modulated by the treatment with etoposide, we performed the experiment using triple SILAC labeling of cells. We compared GFP-based immunoprecipitates from uninduced cells (light, control), doxycycline-induced cells (medium), and doxycycline-induced cells that were treated with etoposide for 1 h, washed and allowed to recover for 15, 60, and 240 min (heavy) (Fig. 4*A*). This experimental design allows quantification of interactions that are increased or decreased over time during the cellular response to DNA damage. The levels of GFP-MCM2 immunoprecipitated were similar in each conditions (Fig. 4*B*), and the overall pattern of identification remained similar throughout the experiment (Fig. 4*C*, 4*D*, and supplemental Table S3). All proteins from the MCM complex were identified at each time point with similar stoichiometry, confirming the stability of the complex following the treatment with etoposide (Fig. 4*D*). We were able to identify several proteins interacting with the MCM complex, including nucleophosmin, BAG2, UPP1, and HDAC10. All those interactions did not change following the treatment with etoposide (Fig. 4*D*). Ubiquitin was also identified as a protein enriched, but in this case most likely because one or some proteins of the MCM complex are ubiquitinated. We found four proteins whose interaction with the MCM complex appeared to be modulated following the DNA damage treatment (Fig. 4*D*, supplemental Table S3). Three of those proteins showed an increased in enrichment, including the histone chaperone ASF1A, the putative tyrosine phosphatase Paladin (PALD1) and Importin-7 (IMP7). On the contrary, the Chromodomain helicase DNA binding protein 3 (CHD3) showed a decrease in interaction following the treatment with etoposide.

**Fig. 5.**
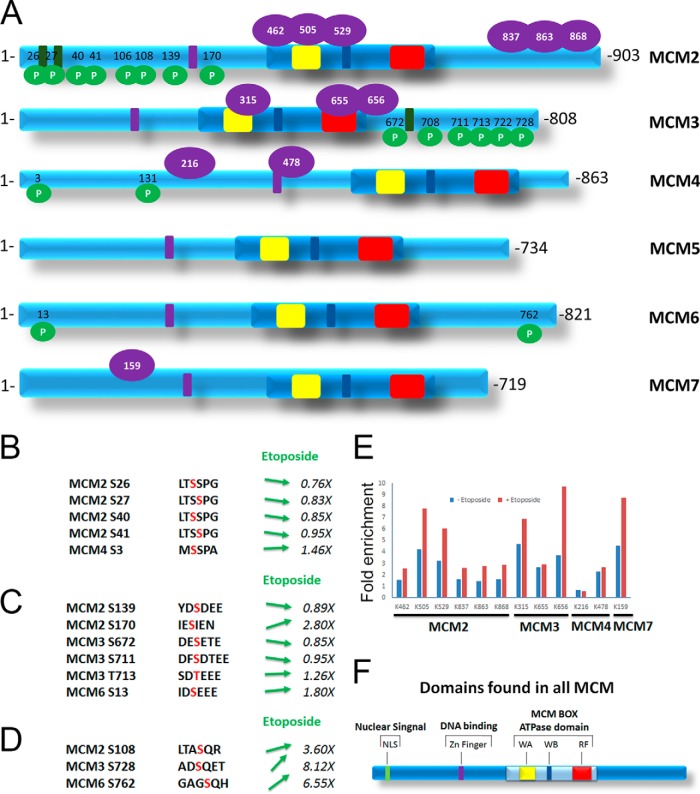
**Identification of post-translation modification on the proteins from the MCM complex.** *A*, Identification of post-translational modifications using either phosphorylation of serine and threonine (green circles) or the presence of a di-glycine peptide (purple circles) as variable modifications indicating extensive modification of the MCM complex. The H/M ratios indicating a change in the presence of the modification following treatment with etoposide is indicated for *B*, serine phosphorylation on SSP motifs, *C*, serine/threonine phosphorylation on (E/D)(S/T)(E/D)EE motifs and *D*, serine phosphorylation of SQ motifs. *E*, The presence of ubiquitinated peptides of MCM proteins from mock treated cells M/L (blue) and cells treated with 50 μm etoposide for 1 h H/L (red) ratios, quantifying the abundance of diglycine peptide signature of ubiquitin-like modifications on the MCM proteins. *F*, Domains found in the MCM proteins as shown in A include the nuclear localization signal (NLS), the zinc finger (Zn Finger) as well as the ATPase domain with the Walker A, B and the arginine finger (RF).

Additionally, it was possible to detect post-translational modifications on the immunoprecipitated proteins, indicating extensive modification of the MCM complex by phosphorylation (Fig. 5*A*, green circles). A closer analysis of the phosphorylation sites allowed us to group the phosphorylation following three distinct consensus sites (Fig. 5*B*, 5*C*, and 5*D*). The SSP motif is known to be phosphorylated on the MCM proteins by the Cdc7/Dbf4 complex, which is responsible for allowing initiation of DNA replication by activation of the MCM complex (29). Our data did not show any increases in the phosphorylation at these sites, indicating that the treatment with etoposide did not activate initiation of DNA replication or synchronization of cells in S phase (Fig. 5*B*). The (E/D)(S/T)(E/D)EE motif corresponds to the canonical phosphorylation motif of casein kinase II (CKII), a kinases with pleiotropic functions that has also been implicated with regulating DNA replication (30). Again, most of these sites did not show any increase in phosphorylation (Fig. 5*C*). Interestingly, three phosphorylation sites, one on each of the MCM2, MCM3, and MCM6 proteins followed the SQ consensus sites for the DNA repair signaling kinases ATM, ATR, and DNA-PK (31, 32) and were found to be increased (Fig. 5*D*), suggesting that the DNA damage induced by the treatment with etoposide specifically resulted in phosphorylation of the MCM complex (Fig. 5*D*). Finally, because ubiquitin was identified as an interacting protein with the MCM complex, the presence of a diglycine peptide signature of ubiquitin modifications was investigated (33–35). It was possible to identify 12 ubiquitination sites on the MCM proteins (Fig. 5*A*, purple circle). The quantification of this modification following the treatment with etoposide showed that ten of the 12 possible ubiquitination sites were up-regulated upon DNA damage (Fig. 5*E*).

**Fig. 6.**
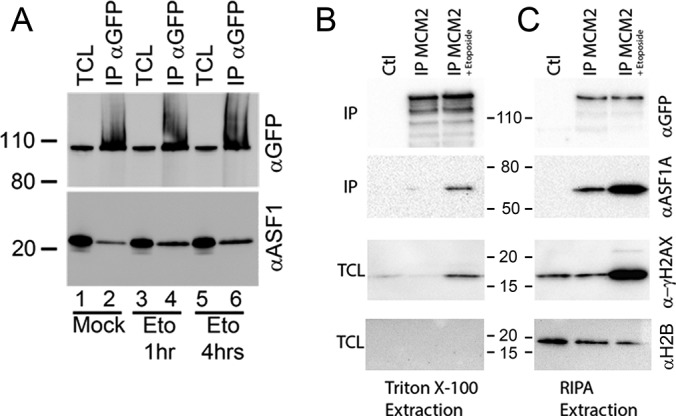
**Interaction between ASF1 and MCM2 is increased following DNA damage.** *A*, U2OS cells expressing GFP-MCM2 were either mock treated (lane 1–2) or treated with etoposide at 50 μm for 1 h and allowed to recover for either 1 h (lane 3–4) or 4 h (lane 5–6). Total cell extracts were immunoprecipitated with GFP-antibodies and immunoblotted with GFP (top) or ASF1A (bottom) antibodies. A similar experiment was performed, but instead comparing protein lysates prepared using either 1% triton X-100 buffer (*B*) or RIPA buffer (*C*). Immunoprecipitates (IP) were immunoblotted with antibodies recognizing GFP or ASF1A, and total cell lysates (TCL) were immunoblotted with antibodies recognizing histone H2AX phosphorylated on serine 139 and histone H2B.

To confirm the changes in interactions observed following DNA damage (Fig. 4*D*), immunoprecipitations of GFP-MCM2 were performed in either mock treated cells, or cells treated with etoposide for 1 h, washed and allowed to recover for 1 and 4 h (Fig. 6). Proteins were separated by SDS-PAGE and immunoblots of total cell lysates with GFP and ASF1 antibodies did not show any change in the total amount of protein present (Fig. 6*A*, lane 1, 3, and 5). However, an increased in the amount of ASF1 was observed (Fig. 6*A*, compare lane 2, 4, and 6), confirming the increase in interaction between the MCM complex and ASF1. To confirm the presence of DNA damage, phosphorylation of H2AX on serine 139 (γH2AX) was assessed with and without treatment with etoposide (Fig. 6*B*, TCL). Treatment with etoposide indeed caused an increase in phosphorylation (Fig. 6*B*, lane 3 *versus* lane 1 and 2). However, the amount of histones present was low as shown by the relatively weak signal obtained (Fig. 6*B*). This could be explained by the extraction method using a 1% triton-X100 lysis buffer, which may not allow a strong extraction of chromatin proteins. Thus, we decided to use a more stringent extraction method using RIPA buffer. When comparing the amount of histones H2AX extracted between the two conditions (Fig. 6*B* and 6*C*, TCL), it appeared that RIPA was able to solubilize a larger amount of histones. To determine whether the interaction between the MCM complex and ASF1 could be associated with chromatin, MCM2 was immunoprecipitated from either mock-treated cells, or cells treated with etoposide for 1 h using either triton X-100 or RIPA buffer. Immunoblots with a GFP antibody shows that the majority of MCM2 was found in the triton X-100 extraction (Fig. 6*B*). However, the interaction with ASF1 appeared to be predominantly found when the cells were extracted with RIPA buffer (Fig. 6*C*), suggesting that although most of the MCM complex is not necessarily associated with chromatin, the association between ASF1 and MCM2 appears to be.

**Fig. 7.**
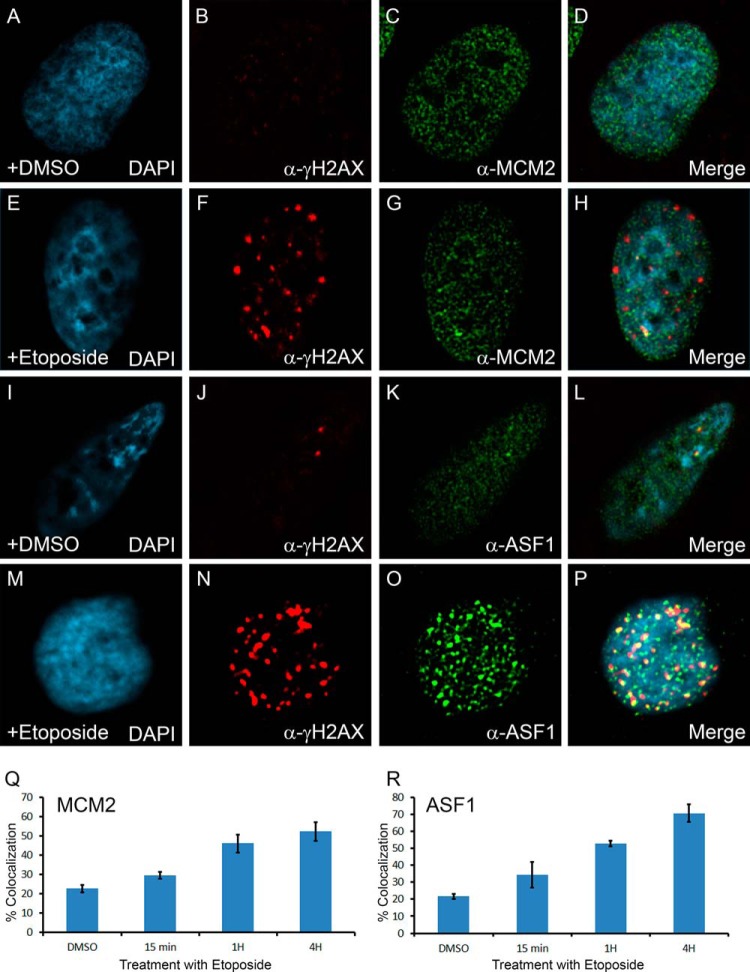
**Co-localization of MCM2 and ASF1 with sites of DNA damage.** U2OS cells grown on glass coverslips were either mock treated (*A–D*, *I–L*) or treated with etoposide at 1 μm for 1 h (*E–H*, *M–P*) prior to fixation with paraformaldehyde and labeled for immunofluorescence with a γH2AX antibody (*B*, *F*, *J*, *N*), a MCM2 antibody (*C*, *G*) or a ASF1 antibody (*K*, *O*). The merge images are shown (*D*, *H*, *L*, and *P*). Percentage of cells with three or more obvious co-localization foci at different time of recovery following treatment with etoposide for both MCM2 (*Q*) and ASF1 (*R*) (more than 100 cells per time point, *n* = 3).

To test whether the interaction between the MCM complex and ASF1 was associated with sites of DNA damage, we assessed the localization of MCM2 and ASF1 in cells before and after DNA damage. DNA damage was induced with etoposide, but with a smaller dose then used in the protein interaction experiments because the signal for γH2AX was too high and undefined in the cells at those higher doses. In the absence of DNA damage, MCM2 and ASF1 were detected throughout the nucleus, but excluded from the nucleolus in the absence of DNA damage (Fig. 7*C* and 7*K*), with little to no signal for γH2AX (Fig. 7*B* and 7*J*). Following treatment with etoposide for 1 h, a large increase in the signal for γH2AX was observed (Fig. 7*F* and 7*N*). Interestingly, we found some partial co-localization of MCM2 with γH2AX (Fig. 7*H*) in some of the foci, that was present in some of the cells. ASF1 showed an even greater effect on its co-localization with γH2AX following the treatment with etoposide (Fig. 7*P*). Because not all cells showed a colocalization, we counted the percentage of cells with three or more obvious colocalization foci at different time of recovery following treatment with etoposide for both MCM2 and ASF1 (more than 100 cells per time point, three independent experiments). The number of foci was selected to avoid counting random events. In both cases, we found a significant increase in the colocalization of the protein with γH2AX, confirming the presence of these proteins to sites of DNA damage.

## DISCUSSION

Our data quantifying changes in protein subcellular localization following treatment with etoposide identified proteins that may play a role in the cellular response to DNA damage. The specificity of the effect observed is underlined by the fact that most of the proteome was not affected by the etoposide treatment, but only a small subset of proteins (93 of 1933, less than 5%). Of the proteins identified, several are already known to play a role in DNA repair, confirming that our approach allows the identification of proteins involved in this process. Moreover, the concomitant identification of proteins with similar redistribution provides clues in potential protein interactions or possible common functional mechanisms in which they are involved. We have found known proteins involved in DNA replication clustered with a similar redistribution profile with proteins that were annotated with nucleotide excision repair and nonhomologous end joining, suggesting possible mechanisms linking DNA replication and DNA repair. Of the proteins identified involved in DNA replication, the six proteins forming the MCM complex were identified.

The interaction study of the MCM proteins allowed the identification of previously known proteins, as well as new interactions. It is interesting to note that while GFP-MCM5 was expressed at near endogenous levels and showed its expected, mainly nuclear distribution, it was not incorporated in a fully functional MCM complex and we failed to identify most of the known interactors. This underlines the limitations of affinity-purification mass spectrometry experiments even when routinely used controls (apparent molecular weight, level of expression, cellular localization) show normal protein behavior and the importance of using multiple approaches before making final conclusions regarding protein interaction network data coming from these proteomic experiments. Another interesting observations is the identification of the MCM binding proteins MCMBP as interacting with MCM2. This was unexpected considering that it was initially proposed that MCMBP could replace MCM2, which would lead to the formation of a different complex with MCM3 to 7 (36). Our results clearly show that MCM2 and MCMBP can be present in the same complex since MCMBP was present when MCM2 was immunoprecipitated.

Four proteins whose interaction with the MCM complex appeared to be modulated following the DNA damage treatment were identified. Three of those proteins showed an increased in enrichment, including ASF1A, Paladin (PALD1) and Importin-7. Paladin is an uncharacterized protein with a putative STY phosphatase domain with no known function. Considering that the MCM complex is phosphorylated on multiple sites, there is an interesting possibility that MCM proteins may be a substrate of Paladin. CHD3 is an ATP-dependent chromatin remodeler member of the class II of CHD proteins, which are characterized by the lack of identifiable DNA-binding domains. CHD3 is a component of the histone deactylase NuRD complex which participates in the compaction of chromatin by deacetylating histones and has been found to be involved in nucleosome remodeling during the DNA double-strand break response (37). Depletion of CHD3 has been shown to results in greater phosphorylation of H2AX and an increase in cell cycle checkpoint sensitivity, demonstrating a role for chromatin compaction in the propagation of DNA damage signaling (38). Interestingly, CHD3 has also been shown to be recruited to sites of DNA damage (39). ASF1 is a histone chaperone that facilitates import of histones into the nucleus (40) and is involved in depositing histone on chromatin during DNA replication (41). Our results are in agreement with the previous identification of an MCM-ASF1 complex associated with histones (42), and our data demonstrate that this interaction is modulated in response to DNA damage. This suggests that additional MCM-ASF1 complexes are assembled following DNA damage, which could be involved in chromatin assembly at sites of DNA damage. This could be coordinated with the decrease in interaction of the MCM complex with CHD3, involved in the opposite function of compacting chromatin. These changes in interactions observed was not the results of the activation of DNA replication or the arrest of cells in S-phase, as we did not observed any increase in phosphorylation on the MCM proteins at sites known to be involved in activation of the DNA replication fork. Furthermore, the time points used are most likely too short for any significant cell cycle synchronization to occur. On the other hand, phosphorylation of MCM proteins on consensus sites that are generally considered a hallmark of DNA damage response proteins were found increased, suggesting a mechanism regulating the MCM complex in response to DNA damage.

Ubiquitination is a post-translational modification that can target protein for degradation, but has also been shown to regulate cellular processes such as cell-cycle, apoptosis, and DNA repair by targeting specific regulatory proteins (43). The proteasome has been involved in the regulation of several DNA repair pathways such as the nucleotide excision repair and homologous recombination (reviewed in (44)). We have identified by mass spectrometry a total of 12 ubiquitination sites distributed on several proteins of the MCM complex, notably MCM2, MCM3, MCM4, and MCM7. Of those, several sites were significantly increased following DNA damage. Interestingly, we identified the proteins DDB1 and CUL4 showing a similar redistribution following DNA damage (Fig. 1), as well as interacting with the MCM-ASF1 complex following DNA damage when performing AP-MS of ASF1 (data not shown). DDB1 is the large subunit (p127) of the heterodimeric DNA damage-binding (DDB) complex while DDB2 (p48) forms the small subunit. This protein complex functions in nucleotide-excision repair and binds to DNA damage. Defective activity of this complex causes the repair defect in patients with xeroderma pigmentosum complementation group E (XPE)—an autosomal recessive disorder characterized by photosensitivity and early onset of carcinomas. The protein encoded by this gene also functions as an adaptor molecule for the cullin 4 (CUL4) ubiquitin E3 ligase complex by facilitating the binding of substrates to this complex and the ubiquitination of proteins. It is thus possible that the MCM complex is a target for ubiquitination by the DDB/CUL4 ubiquitin ligase.

In summary, we have identified several proteins associated with MCM proteins that may provide insight into its role in chromatin remodeling and histone deposition following DNA damage, for which little biochemical data are currently available. We used an affinity-purification coupled to mass spectrometry based approach to not only identify interaction partners of the MCM complex, but we have also measured the differences in interactions following DNA damage over time. These experiments represents an additional level of complexity to MS-based proteomics that not only identifies proteins but also provides an unbiased and quantitative measurement of the dynamics of protein interaction networks. This information provides insights in protein complexes from cells under specific conditions.

## Supplementary Material

Supplemental Data

## Footnotes

^1^ The abbreviations used are:

MCM: minichromosome maintenance
SILAC: stable isotope labeling with amino acids in cell culture
GFP: green fluorescent protein
MCMBP: MCM binding protein.
