# Supplementary material for: Quantitative Proteomics Reveals Dynamic Interactions of the Minichromosome Maintenance Complex (MCM) in the Cellular Response to Etoposide Induced DNA Damage
Source: Mol Cell Proteomics. 2015 May 11;14(7):2002–13. doi: 10.1074/mcp.M115.048991 (PMC4587322; doi:10.1074/mcp.M115.048991)
Supplement: Supplemental Data [file supp_14_7_2002__index.html]

Quantitative Proteomics Reveals Dynamic Interactions of the Minichromosome Maintenance Complex (MCM) in the Cellular Response to Etoposide Induced DNA Damage — A Role for the MCM Complex in DNA Repair — Supplemental Data 

# Quantitative Proteomics Reveals Dynamic Interactions of the Minichromosome Maintenance Complex (MCM) in the Cellular Response to Etoposide Induced DNA Damage

## Supplemental Data

- Supplementary Figure Legends - Supplementary Figure Legends
- Supplementary Table 1 - Supplementary Table 1
- Supplementary Table 2 - Supplementary Table 2
- Supplementary Table 3 - Supplementary Table 3
- Supplementary Table 4 - Supplementary Table 4
- Supplementary Figures - Supplementary Figures
