# Supplementary material for: Quantitative Proteomics Reveals Dynamic Interactions of the Minichromosome Maintenance Complex (MCM) in the Cellular Response to Etoposide Induced DNA Damage
Source: Mol Cell Proteomics. 2015 May 11;14(7):2002–13. doi: 10.1074/mcp.M115.048991 (PMC4587322; doi:10.1074/mcp.M115.048991)
Supplement: Supplemental Data [file supp_M115.048991_mcp.M115.048991-1.pdf]

SUPPLEMENTARY DATA

Supplementary Table 1

Table 1a : List of proteins from the spatial proteomics analysis found in Figure 1B with the medium (M) label represents the nucleus and the light (L) label represents the cytoplasm.

Table 1b: List of the 93 proteins that shows a change in localization in their nucleo-cytoplasmic ratios as shown in Figure 1D.

Table 1c: List of proteins sorted by KEGG pathway enrichment as shown in Figure 1C.

Table 1d: List of proteins as shown in the table of Figure 1E.

Supplementary Table 2

Tables with data presented in Figure 3. GFP tagged MCM2 and MCM5 proteins were immunoprecipitated using GFP-TRAP agarose beads, ensuring a near depletion of all the tagged MCM protein from the cell lysate. C) SILAC-labelled cells were used for comparison of GFP-based immunoprecipitates from uninduced cells (light) with doxycycline-induced cells (medium). Immunoprecipitates were combined, separated by SDS-PAGE, each gel lane cut into 8 slices prior to in-gel digestion with trypsin. The extracted peptides were analysed by LC-MS/MS. The M/L ratio of two independent experiments for each of GFP-MCM2 (Table MCM2) and GFP-MCM5 (Table MCM5) are presented.

Supplementary Table 3

Table with the data presented in Figure 4. SILAC-labelled cells were used for comparison of GFP-based immunoprecipitates from uninduced cells (light), of DMSO (mock) treated cells induced for GFP-MCM2 expression (medium) and of etoposide treated cells induced for GFP-MCM2

expression (heavy). Ratios of proteins interacting with GFP-MCM2 from mock treated cells (M/L) versus cells treated with etoposide for 1 hour at 50 μM and allowed to recover for 15, 60 and 240 minutes (H/L).

Supplementary Table 4

Table with the data presented in Figure 5. Identification of post-translational modifications using either phosphorylation of serine and threonine or the presence of a di-glycine peptide as variable modifications indicating extensive modification of the MCM complex. The H/M ratios indicating a change in the presence of the modification following treatment with etoposide.

Supplementary Figure 1

Annotated mass labeled spectra and MS/MS spectra for a non-proline containing peptide (LQAEIEGLK, A and B), and a proline containing peptide (LLLPGELAK, C and D) showing minimal arginine to proline conversion (<5% heavy proline peak at 484.3).

Supplementary Figure 2

A) Whole cell extracts from either non-induced U2OS cells (lanes 1 and 4), or from cells induced for expression of GFP-MCM2 with increasing concentrations of doxycycline (lanes 2, 3, 5 and 6) were separated by SDS-PAGE and immunoblotted with a GFP antibody (lanes 1-3), or with a MCM2 antibody (lanes 4-6) to confirm expression of the GFP-tagged proteins.

Supplementary Figure 3

A) U2OS (lanes 1, 4 and 7), or U2OS cells expressing GFP-MCM2 were either mock treated (lanes 2, 5 and 8) or treated with etoposide at 50 μM for 1 hour and allowed to recover for 1 hour (lanes

3, 6 and 9). Total cell extracts were immunoprecipitated with GFP-Trap agarose beads (lanes 1-3 and 7-9) and immunoblotted with MCM5 (lanes 1-3) and GFP (lanes 7-9) antibodies. Total cell lysates (lanes 4-6) were immunoblotted with a MCM5 antibody. **B**) U2OS (lanes 1, 4 and 7), or U2OS cells expressing GFP-MCM5 were either mock treated (lanes 2, 5 and 8) or treated with etoposide at 50 μM for 1 hour and allowed to recover for 1 hour (lanes 3, 6 and 9). Total cell extracts were immunoprecipitated with GFP-Trap agarose beads (lanes 1-3 and 7-9) and immunoblotted with MCM2 (lanes 1-3) and GFP (lanes 7-9) antibodies. Total cell lysates (lanes 4-6) were immunoblotted with a MCM2 antibody.
