# Supplementary figures and images for: Quantitative Proteomics Reveals Dynamic Interactions of the Minichromosome Maintenance Complex (MCM) in the Cellular Response to Etoposide Induced DNA Damage
Source: Mol Cell Proteomics. 2015 May 11;14(7):2002–13. doi: 10.1074/mcp.M115.048991 (PMC4587322; doi:10.1074/mcp.M115.048991)

Supplementary Figure 1

A

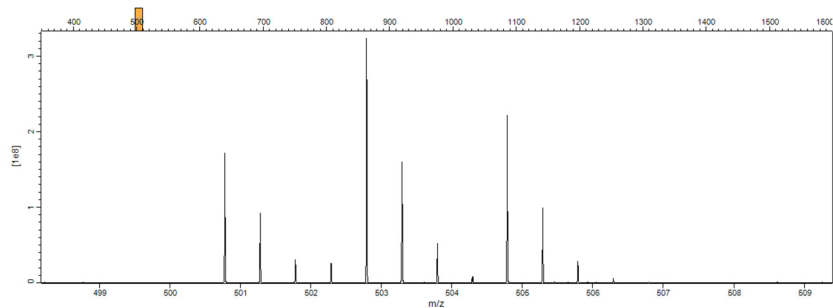

B

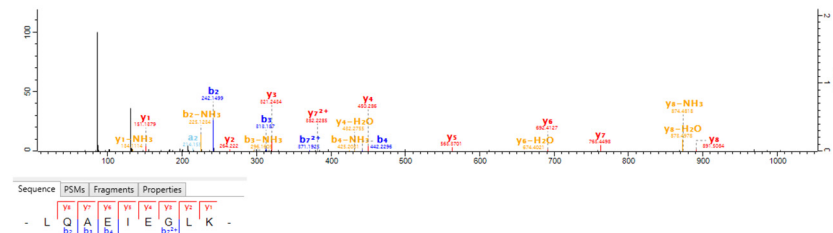

C

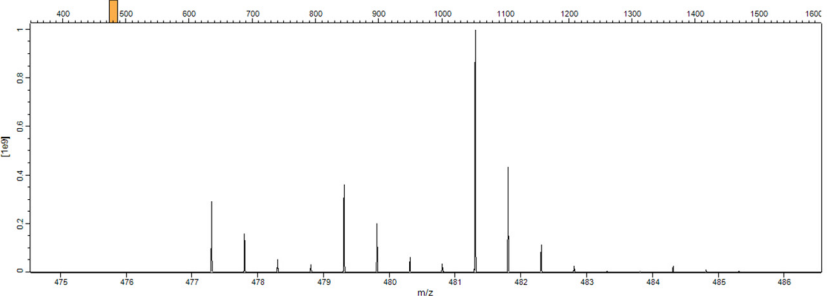

D

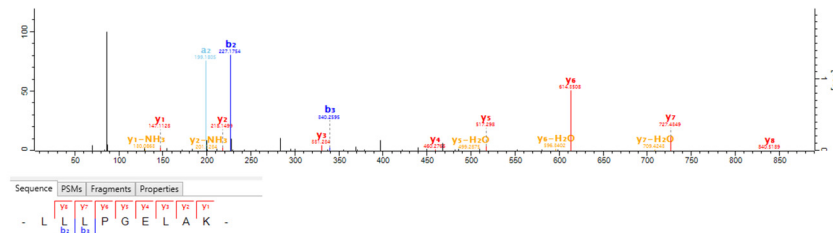

Supplementary Figure 2

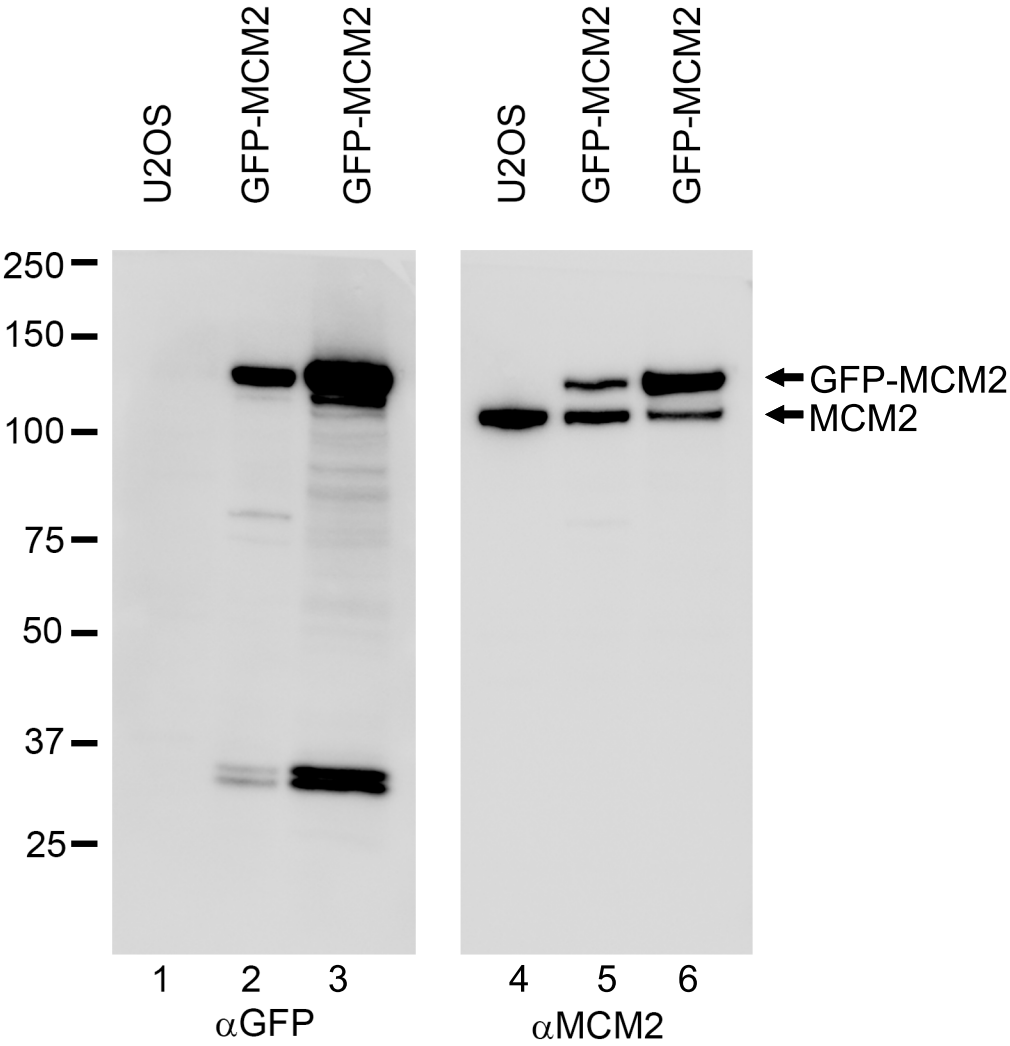

Supplementary Figure 3

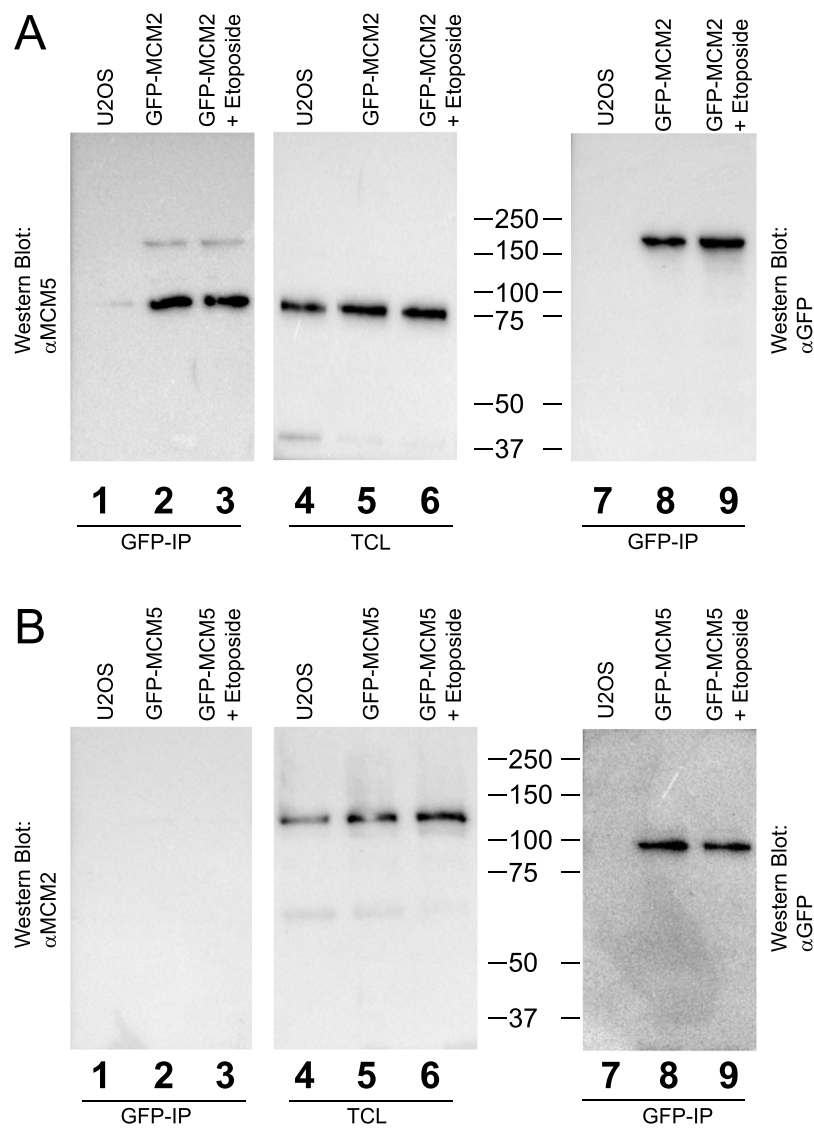

Supplement: Supplemental Data [file supp_M115.048991_mcp.M115.048991-6.pdf]
